# Consequences and impacts of PEG-IFNα2a shortage: first lessons from a MPN French center

**DOI:** 10.1007/s00277-025-06510-y

**Published:** 2025-07-17

**Authors:** Laura Cailly, Wayne-Corentin Lambert, Jean-Richard Eveillard, Brigitte Pan-Petesch, Laura Herbreteau, Laetitia Rio, Lanig Civi, Adrian Tempescul, Florence Dalbies, Hussam Saad, Cristina Bagacean, Marie-Anne Couturier, Gaelle Guillerm, Eric Lippert, Jean-Christophe Ianotto

**Affiliations:** 1 Service d’Hématologie et d’Hémostase Clinique, Hôpital de la Cavale Blanche, CHU de Brest, Bld Tanguy Prigent, Brest cedex, 29609 France; 2 Laboratoire d’Hématologie, CHU de Brest, Brest, France; 3 Univ Brest, INSERM, GETBO, Brest, UMR1304 France; 4 France Intergroupe des Néoplasies Myéloprolifératives, Paris, France

**Keywords:** Myeloproliferative neoplasms, PEG-IFNα2a, Shortage, Outcomes

## Abstract

Between March to December 2024, we observed a PEG-IFNα2a shortage in France for MPN patients. We identified and followed 90 patients from our center and observed 80% of difficulties obtaining the drug, and 61 patients among all (67.8%) did not receive their prescribed drug on time (impacted group). Twenty-one patients need to reduce the dosage of PEG-IFNα2a and 27 stopped the drug. Ten clinical events were identified with nine in the impacted group (*p*=0.04). No hematological or only partial responses were more frequent in the impacted group (*p*=0.01). The allelic burden variations were also studied during this period; 17 patients obtained a VAF <10%. Eight patients stopped the drug because of VAF <4% and remained with molecular response. Three-quarters of the patients experimented with psychological and financial impacts of shortage. This study is the first one to explore a shortage of cytoreductive drugs happened in MPN. Much informations will be helpful for clinicians.

**Supplementary Information:**

The online version contains supplementary material available at 10.1007/s00277-025-06510-y.

Dear Editor-in-Chief,

Philadelphia-negative myeloproliferative neoplasms (MPN) are characterised by deregulated proliferation of myeloid cells, mainly platelet and erythrocyte progenitors. Thrombotic events are the most common adverse events, justifying antithrombotic treatment and cytoreductive therapy in “high thrombotic/hemorrhagic risk” patients. The aim of cytoreductive therapies is to normalise blood counts to reduce the thrombotic/hemorrhagic risk [1–3]. Recent studies seem to indicate that reducing the disease burden may also reduce the risk of long-term complications, including progression to myelofibrosis [4]. Among these, PEG-IFN has demonstrated remarkable efficacy in reducing the JAK2V617F or CALR exon 9 mutation allele burden [5, 6]. It is therefore the treatment of choice in the first line setting for young patients and typically in second line for elderly patients [7]. Unfortunately, due to difficulties in the production of PEG-IFNα2a at the beginning of March 2024, the only PEG-IFN then available in France, many patients have had difficulties in ensuring the continuity of their treatment. In September 2024, PEG-IFNα2b (ROPEG-IFN) has become available, but with the limited indication of starting treatment in PV patients (without symptomatic splenomegaly) [8]. In the absence of information on this difficult treatment period, we investigated the real outcomes and impact of the PEG-IFNα2a shortage in our MPN population.

As of March 1 st, 2024, 90 patients (15.8% of the treated cohort) of our monocentric OBENE cohort (NCT02897297), which prospectively collects information on MPN patients, received PEG-IFNα2a alone (85.6%) or in combination with another cytoreductive drug (14.4%) (Supplementary Fig. 1). Of these, 41 (45.6%) had polycythemia vera (PV), 29 (32.2%) had essential thrombocythemia (ET) and 20 (22.2%) had primary or secondary myelofibrosis (MF) or unclassified MPN. The *JAK2*V617F mutation was identified in 70 patients (77,8%). PEG-IFNα2a was prescribed as first line (29 pts), second line (36 pts) or further (25 pts). The median time on PEG-IFNα2a was 5.7 years (0.1–19 years). The median starting dose of PEG-IFNα2a was 33.8 µg/wk (9-135) (Supplementary Table 1).

The observation period was from March 1 st to December 31 st, 2024. At the end of December, 72 patients (80%) had had difficulties obtaining the drug from their local pharmacy and 61 patients (67.8%) did not receive their prescribed treatment on time (“impacted” group) (Table 1 and Supplementary Table 1). Delays ranged from one week to several months. This induced many modifications of the cytoreductive drug regimens (Fig. 1 and Supplementary Fig. 2): 63 patients (70%) were maintained with PEG-IFNα2a; 55 patients (62.2%) had at least one modification of their drug (38/69.1% from the “impacted group”, *p* = 0.05 compared to “non-impacted group”); 5 had increased dosage (median weekly dose from 22.5 to 33.8, all “impacted”) and 3 others were prescribed an additional cytoreductive drug (2 Anagrelide, 1 Hydroxyurea, all “impacted”); 21 had decreased dosage (15 “impacted”-66.6%, 45 to 22.5 µg). Twenty-seven patients had stopped PEG-IFNα2a (16 “impacted”, 59.3%). Phlebotomies were performed to reduce hematocrit in 12 PV patients (29.3%) (7 “impacted”-58.3%) (Table 1).

**Fig. 1:**
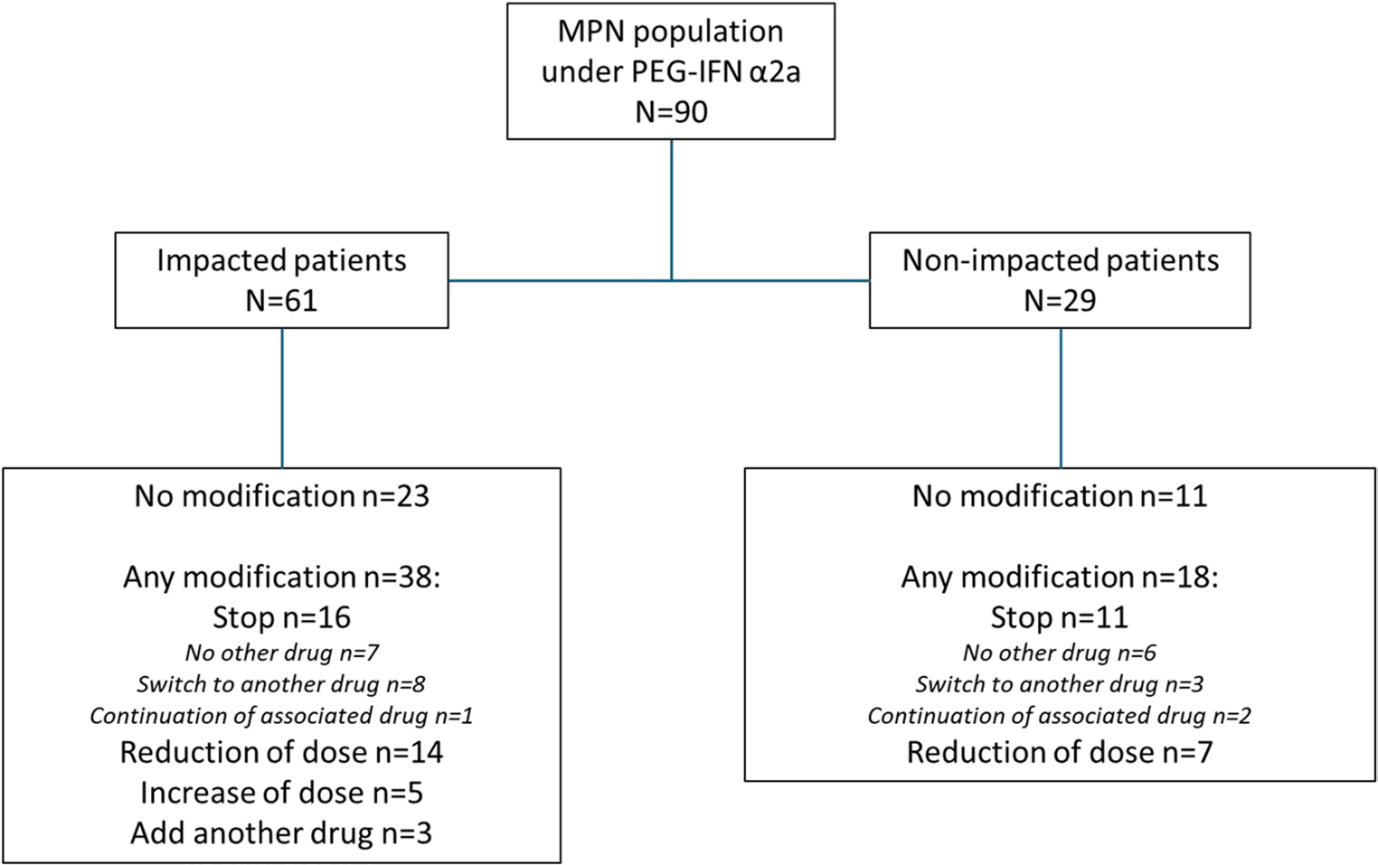
Evolution of treatments according to the impacted and non-impacted status

**Table 1:** Consequences of PEG-IFNα2a shortage in the impacted MPN group. CR=complete hematological response; PEG: PEG-IFNα2a

	All	Impacted population	Non-impacted population
Numbers	90	61	29
Evolution of prescription			
No-change of drug	35	23	12
Any change of drug	55	38	17
Increased dose of PEG	5	5	0
Added drug	3	3	0
Decreased dose of PEG	21	15	6
Stopped PEG	27	16	11
New phlebotomies	12	7	5
Events during the study			
Clinical events	10	9	1
Hematological responses			
No CR	18	16	2
Loss of CR	6	3	3
Maintain of CR	49	31	18
Obtention of CR	17	11	6

During follow-up, ten cardiovascular or evolutive events were identified: 4 thromboses, 2 hemorrhages (1 death from cerebral hemorrhage), 2 phenotypic evolutions, 2 hyperviscosity-related cardiac complications (grade 4 hypertension and atrial fibrillation) (Supplementary Table 2). Nine of these 10 events occurred in “impacted” patient group (OR = 6.89 [0.88;314.95], *p* = 0.04). During the 1-year period before the shortage, two events were reported among the 88 treated patients (1 post-PV MF and 1 hemorrhagic pleural effusion) (*p* = 0.03; OR = 5.47 [1.11;52.82], between present study to previous history).

Regarding hematological responses, 57 patients (63.3%) were in complete and 33 (36.7%) partial responses respectively at the beginning of the observation, versus 64 (71.1%) and 26 (28.9%) at the end (+ 7.8% of CR) (Fig. 2). The increased rate of CR was less important in patients who were impacted (+ 5.6 vs. 11%). The proportions of responses are presented in Table 1. Seven of ten clinical events were observed in non-CR patients; two ocular thromboses happened in CR patients (Supplementary Table 2).

**Fig. 2:**
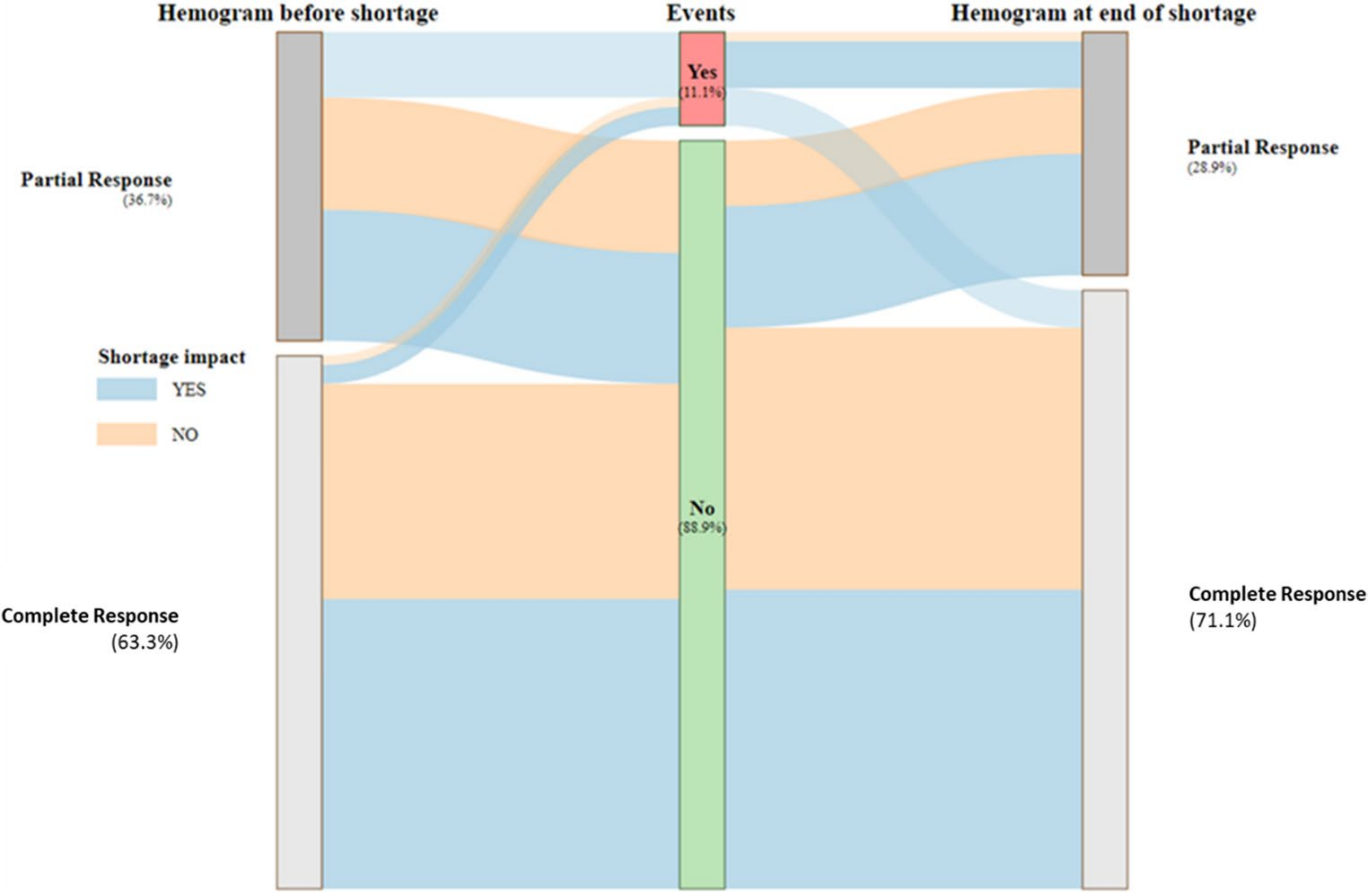
Sankey chart of events and hematological response during the PEG-IFNα2a shortage

We also collected *JAK2*V617F allele burdens (AB) in 57 patients during this period (38 both before and after). The median AB values were 17 [0.01-83%] and 13% [0–86%], respectively. All variations are shown in Supplementary Fig. 3. No significant AB variation was observed during this time, probably due to low numbers of patients (28 and 10, respectively). Despite that, we identified 17/57 patients (29.8%) with low AB (< 10%). According to previous publications, 8 stopped PEG-IFNα2a without change of AB (4 AB ≤ 1% remained ≤ 1%) as a demonstration of PEG-IFN effect reminiscence [10].

Furthermore, 27 patients discontinued PEG-IFNα2a, 11 had no impact of the shortage (40.7%), suggesting that unavailability was not the major cause of discontinuation. Among them, 11/27 switched to another drug (8 impacted) and 3/27 continued the associated drug (1 impacted) (Fig. 1 and Supplementary Figs. 2). Sixteen patients kept or gained a CR, but 11 lost CR or stayed in PR. In eleven patients who were switched to another cytoreduction, only 3 had not been impacted by the shortage (27.2%). Three gained a CR, 3 remained in CR, and 5 remained in PR. The three patients who were switched while having sustained impact of the shortage either gained CR (2) or remained in CR (1). Five events were observed among these patients (*p* = 0.16, OR = 2.6 [1.69;27.85]). Overall, discontinuation of PEG-IFNα2a was associated with more frequent absence or loss of hematological responses (15/27 vs. 18/63, *p* = 0.018, OR = 3.08 [1.11;8.83]) [9, 10].

This long period has been detrimental for most of the patients, affecting their quality of life, increasing psychological distress and altering treatment observance, thus amplifying the PEG-IFN shortage direct impact [11–13]. Seventy patients (77.8%) expressed psychological and financial impacts. Most of the reasons were waiting for doses/delay of injections (60 pts-85.7%), fear of losing hematological responses (50-71.4%), amount of time spent to ask different pharmacies to access drug (25 pts-35.7%), reappearance of clinical symptoms (20 pts-22.2%) and having to drive many kilometers to get the drug (other pharmacies in the same town, other town and area… 10 pts-14.3%). More than 200 phone calls, consultations and email exchanges have been made during this period.

Some biases could be found in this study: low number of patients, short period of follow-up and incomplete molecular evaluation. On the contrary, all the patients were followed from beginning to end; all dosages, drug modifications, hemograms and events have been correctly collected.

This is the first report on outcomes and impacts of PEG-IFNα2a shortage on MPN patients. In these difficult times, when a new period of PEG-IFNα2a shortage is beginning, we identified that the first one was associated with high rates of patient’s impacts and adverse events (clinical or hematological responses) even though we cannot establish a causative link between the shortage and event outcomes. Few patients lost their hematological response because of other cytoreductive drug availability. As clinicians, we have been aware of possible impacts of this situation and screen all patients treated by PEG-IFNα2a to adequately manage their prescriptions. To complete these results, a new prospective study is ongoing, because of the second PEG-IFNα2a shortage.

## Supplementary Information

Below is the link to the electronic supplementary material.

Supplementary file1 (DOCX 198 KB)

## Data Availability

No datasets were generated or analysed during the current study.
