# Supplementary material for: Consequences and impacts of PEG-IFNα2a shortage: first lessons from a MPN French center
Source: Ann Hematol. 2025 Jul 17;104(8):4263–6. doi: 10.1007/s00277-025-06510-y (PMC12432018; doi:10.1007/s00277-025-06510-y)
Supplement: Supplementary file 1 — Supplementary file1 (DOCX 198 KB) [file 277_2025_6510_MOESM1_ESM.docx]

**Supplementary Figure 1.** Flow-chart of the OBENE population at the time of the shortage beginning.

**
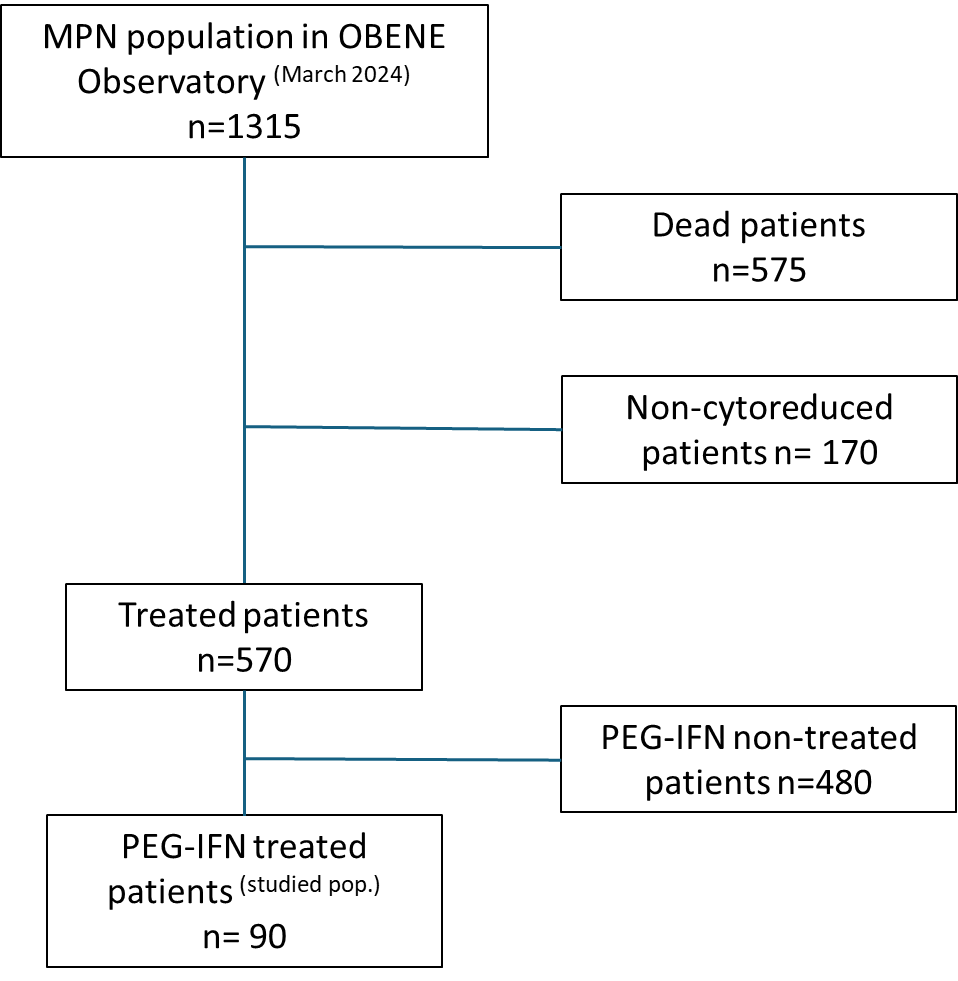
**

**Supplementary Figure 2.** Evolution of treatments during the study period.

**
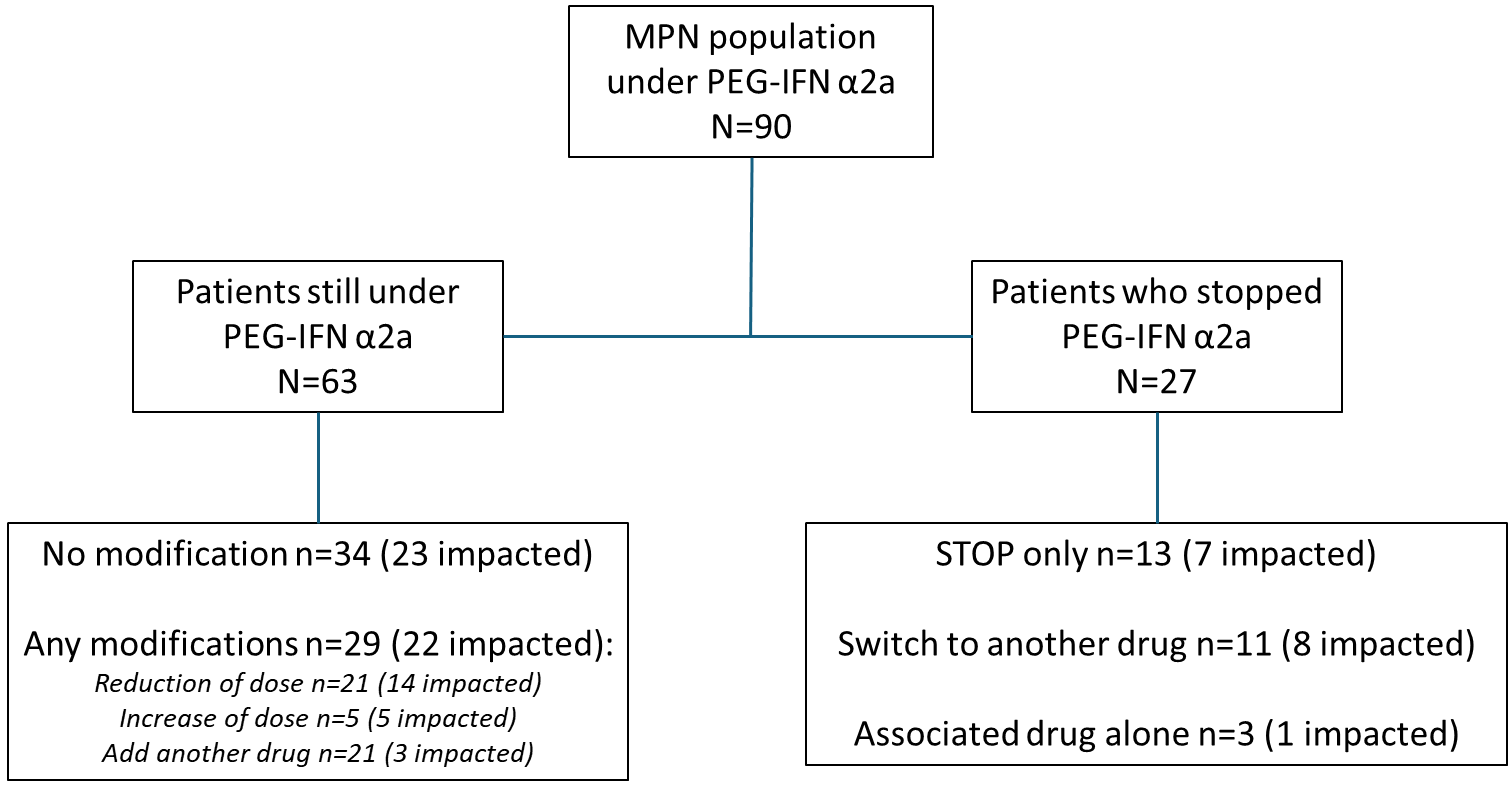
**

**Supplementary Figure 3.** Evolution of JAK2 allele burdens during the period study: Whole cohort (A), impacted patients in red (B) and non-impacted patients in blue (C). *Median expressed in percentage.*

**
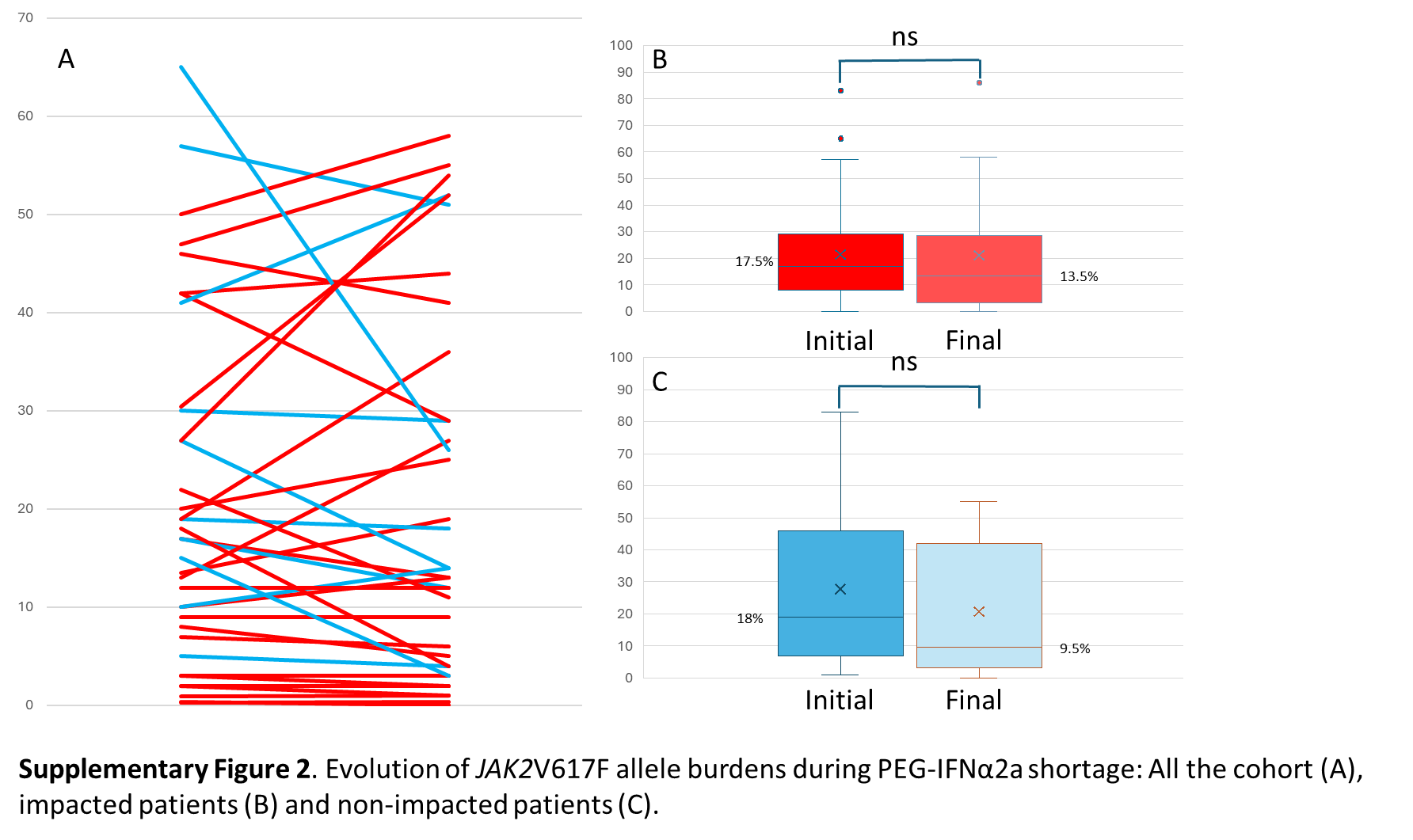
**

**Supplementary Table 1**. Description of the MPN population during PEG-IFNα2a shortage.

**Supplementary Table 2.** Clinical events happening during the PEG-IFNα2a shortage.
